# Development of a Novel Dorsal Incision Only Invagination Type Pancreatogastrostomy (Charité-PG) Following Open Pancreaticoduodenectomy—A Single Centre Experience

**DOI:** 10.3390/jcm10122573

**Published:** 2021-06-10

**Authors:** Lea Timmermann, Marcus Bahra, Johann Pratschke, Thomas Malinka

**Affiliations:** 1 Department of Surgery, Charité-Universitätsmedizin Berlin, Corporate Member of Freie Universität Berlin, Humboldt-Universität zu Berlin, and Berlin Institute of Health, 14195 Berlin, Germany; johann.pratschke@charite.de (J.P.); Thomas.malinka@charite.de (T.M.); 2 Department of Oncological Upper Abdominal Surgery and Robotic- Krankenhaus Waldfriede, 14163 Berlin, Germany; m.bahra@waldfriede.de

**Keywords:** pancreatogastrostomy, pancreato-enteric anastomosis, pancreaticoduodenectomy

## Abstract

The implementation of a pancreatico-enteric anastomosis following open single stage pancreaticoduodenectomy (PD) is still associated with the most threatening complications in modern pancreatic surgery, such as postoperative pancreatic fistula (POPF), postpancreatectomy haemorrhage (PPH), delayed gastric emptying (DGE), intraabdominal abscesses and related mortality. With this study, we introduce Charité-PG, a new dorsal incision only invagination type pancreatogastrostomy (dioPG) for the restoration of the pancreatic remnant following PD, and compare it to a PG requiring ventral gastrotomy (vgPG). A total of 49 consecutive patients, who underwent reconstruction via dioPG, and 92 consecutive patients, who underwent restoration via vgPG, were identified from our prospective database and further reviewed for perioperative parameters, complication rates, mortality and follow-up. The percentage of overall complications (*p* = 0.301), as well as the 30-day mortality rate (*p* = 0.725) and survival (*p* = 0.543), were comparable in both groups. The operation time in the dioPG group was significantly shorter (*p* = 0.04), and patients in this group developed substantially fewer rates of DGE (*p* = 0.036). We provide a feasible and safe technique for restoration following PD via our novel dioPG, causing fewer cases of DGE. Nevertheless, pancreatico-enteric anastomoses require expertise and experience.

## 1. Introduction

The evolution of reconstruction techniques following pancreaticoduodenectomies (PD) started in the late 19th and early 20th century and continued further on. Some general principles to a successful implementation of a pancreatico-enteric anastomosis can be proposed, including the absence of tension in both the pancreatic remnant and the hollow intestine, sufficient blood supply, coverage of the cutting surface and avoidance of necrosis due to tight sutures. The first partial PD was performed in 1898 by Codivilla, who did not restore or ligate the pancreatic stump leading to a massive postoperative pancreatic fistula (POPF) followed by the patient’s death due to malnutrition [1]. In 1912, Kausch restored the pancreatic stump via a pancreaticoduodenostomy after performing his approach to a partial PD [2]. Whipple, who developed the implementation of a pancreaticojejunostomy (PJ), introduced the first one-step procedure in 1946. He inserted a small rubber tube into the pancreatic duct and the jejunum and sewed the parenchyma onto the jejunal wall [3]. Cattell developed the first duct-to-mucosa anastomosis by sewing the pancreatic duct onto the jejunal mucosa requiring a specific duct diameter [4]. Today, a diverse spectrum of methods used for the PJ implementation can be found. A currently widely used technique for PJ is the Blumgart-anastomosis consisting of a two-layered suture with each a duct-to-mucosa and a parenchyma-to-wall anastomosis [5]. In a recent meta-analysis, modified Blumgart-anastomosis appeared to cause fewer severe POPFs and intraabdominal abscesses compared to an interrupted transpancreatic suture [6]. The first pancreatogastrostomy (PG) in a human was performed in 1944 by Waugh and Clagett [7]. Several modifications have been made, and various types of techniques are used today, most commonly including invagination techniques alongside duct-to-mucosa anastomoses. Nevertheless, all modifications that have been made in the last decades aim to reduce the main threats of pancreatic surgery that are highly associated with restoration of the pancreatic stump being, therefore, also referred to as the ‘Achilles’ heel’ of modern single-stage PD [8]. These include the occurrence of POPF, postoperative pancreatic haemorrhage (PPH), delayed gastric emptying (DGE), intraabdominal abscesses as well as overall morbidity and mortality. Different types of open PG have one thing in common; they usually require a ventral gastrotomy regardless of whether an invagination or duct-to-mucosa technique is applied. As minimally invasive and robotic-assisted approaches to a single-stage PD are essential issues in modern pancreatic surgery, we intended to develop an anastomosis feasible also for those approaches. Thus, with this study, we provide a new technique for invagination type PG without ventral gastrotomy, the Charité-Anastomosis (further referred to as dorsal incision only PG; dioPG) and compare it to a classic invagination type PG (ventral gastrotomy PG; vgPG) for outcome parameters such as the incidence of common complications (e.g., POPF, PPH, DGE) as well as mortality-rates and follow-up.

## 2. Materials and Methods

### 2.1. Data Collection and Exclusion Criteria

A retrospective single-centre analysis was performed at our tertiary referral centre for pancreatic surgery. Data of patients who underwent PD with the restoration of the pancreatic remnant via PG in the time between January 2017 and December 2019 were consecutively collected in a database and further reviewed. Exclusion criteria were the performance of other resections than PD, including distal pancreatectomy and total pancreatectomy, reconstruction via PJ and procedures performed laparoscopically or with the use of a robotic system. Thus, an overall of 141 consecutive patients was included. In total, 92 of them underwent reconstruction via vgPG and 49 via dioPG. The following data were evaluated: age, sex, preoperative ASA-score, preoperative BMI, indication, R0-resection state, operation time, overall complications, Clavien/Dindo classification, POPF, PPH, DGE, PG-insufficiency, BDA-insufficiency, surgical site infections (SSI), reoperation rate, intervention rate, in-hospital stay, 30-day mortality and 90-day readmission rate. POPF, PPH and DGE were defined and classified after the International Study Group of Pancreatic Surgery (ISGPS) classifications [9,10,11].

### 2.2. Preoperative Assessment and Preparation

Preoperative assessment included computed tomography with contrast agents or magnetic resonance imaging. In the case of suspected malignancy, the staging was completed by chest imaging and endosonography if indicated. An interdisciplinary tumour board evaluated all cases. Standard preparation included a physical examination, laboratory testing and measuring of CEA and CA 19-9 as well as an individual anaesthesiological risk stratification.

### 2.3. Surgical Approach

Following transverse upper laparotomy and insertion of the retractor-system, the exposed peritoneal cavity is further explored to confirm local resectability and, in case of malignant tumour, absence of peritoneal and hepatic metastasis. Resection starts with retrograde cholecystectomy followed by accessing the omental bursa and preparation of the common hepatic artery to its origin at the celiac axis, combined with a local lymphadenectomy. The Arteria gastroduodenalis is then ligated and dissected. The portal vein is followed to the upper pancreatic margin persecuted by Kocher manoeuvre and stapling of each post-pyloric duodenum and jejunum. The pancreas is then tunnelled at the level of the superior mesenteric vein to the portal vein and dissected with the use of electrocautery. The pancreatic remnant is then mobilised to ensure a tension-free anastomosis by separating it from the V. lienalis and adherent soft tissue. Implementation of vgPG and dioPG is described in the next sections. Reconstruction is completed with the implementation of a hepaticojejunostomy (posterior wall with 5/0 PDS**^®^** (Ethicon, Johnson & Johnson, New Brunswick, NJ, USA) continuous suture and anterior wall with 5/0 PDS**^®^** single button sutures) and an antecolic gastrojejunostomy. Pancreatic and DHC cutting margins, as well as lymphatic tissue from the upper pancreatic margin, undergo fast-frozen sectioning to examine the necessity of further resection.

### 2.4. Reconstruction Following PD via vgPG

**Figure 1:**
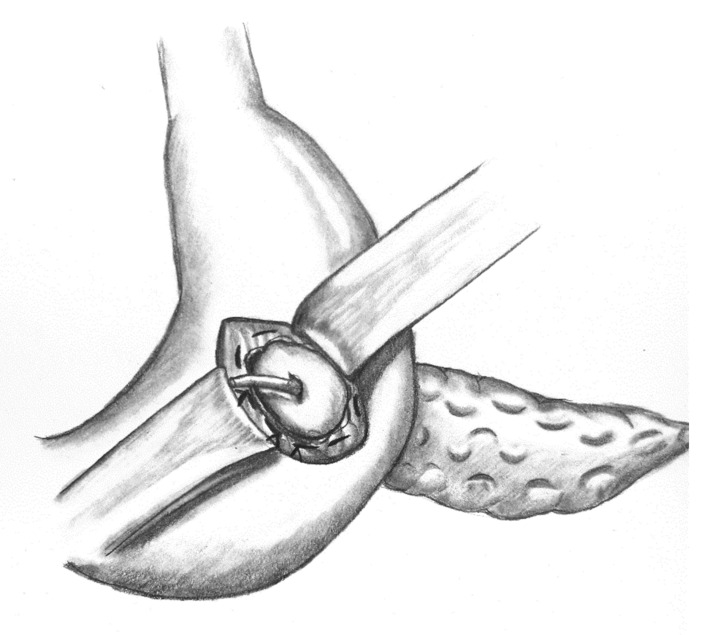
Implemented vgPG following PD.

Initially, an oblique incision to the posterior gastric wall is performed followed by a ventral gastrotomy. A purse-string suture is then placed around the dorsal incision using Prolene**^®^** 4/0, and the pancreatic remnant is luxated through the posterior gastric wall. A small rubber tube is inserted into the pancreatic duct and secured with PDS**^®^** 5/0. Three mattress sutures (PDS**^®^** 4/0) are then placed using MH 1 needle. Mattress sutures and consecutively the purse-string suture are tied. Ventral gastrotomy is closed with a double-layered PDS**^®^** 4/0 suture. Figure 1 shows the implemented PG before the closure of ventral gastrotomy. A closed suction-type and tubulo-laminar type drainage are positioned at the anastomosis and resection site.

### 2.5. Reconstruction Following PD via dioPG (“Charité-PG”)

**Figure 2:**
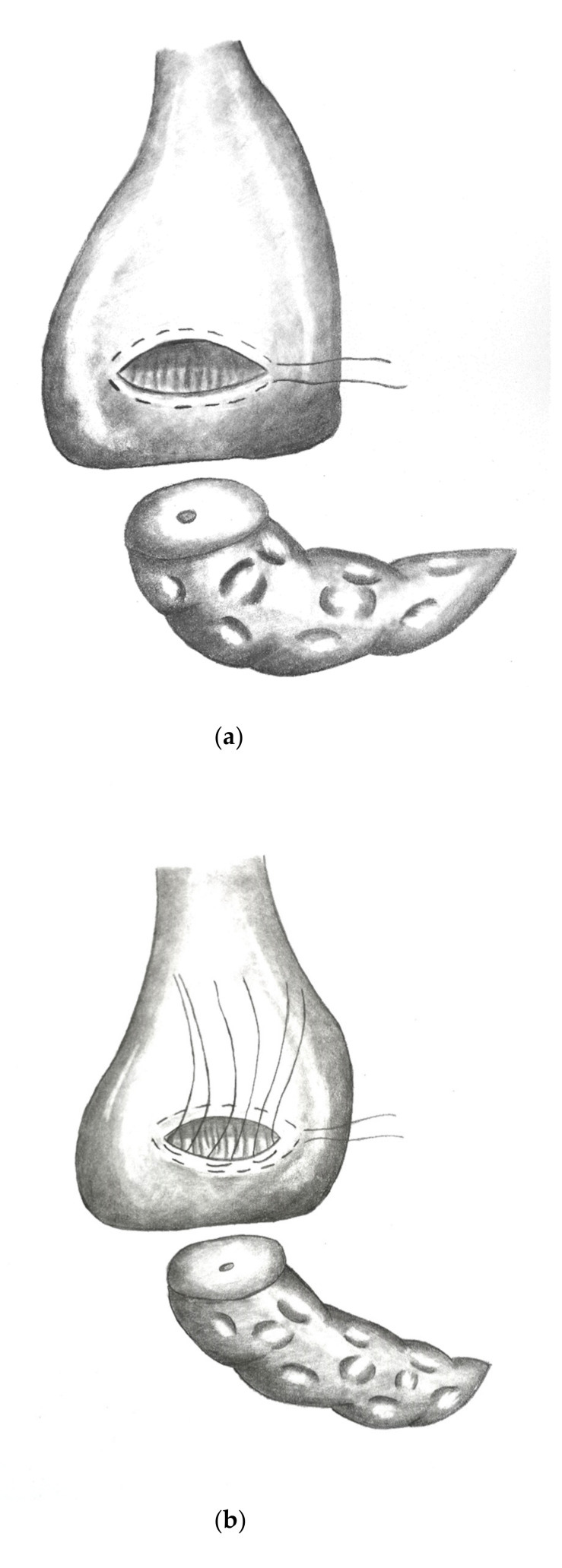

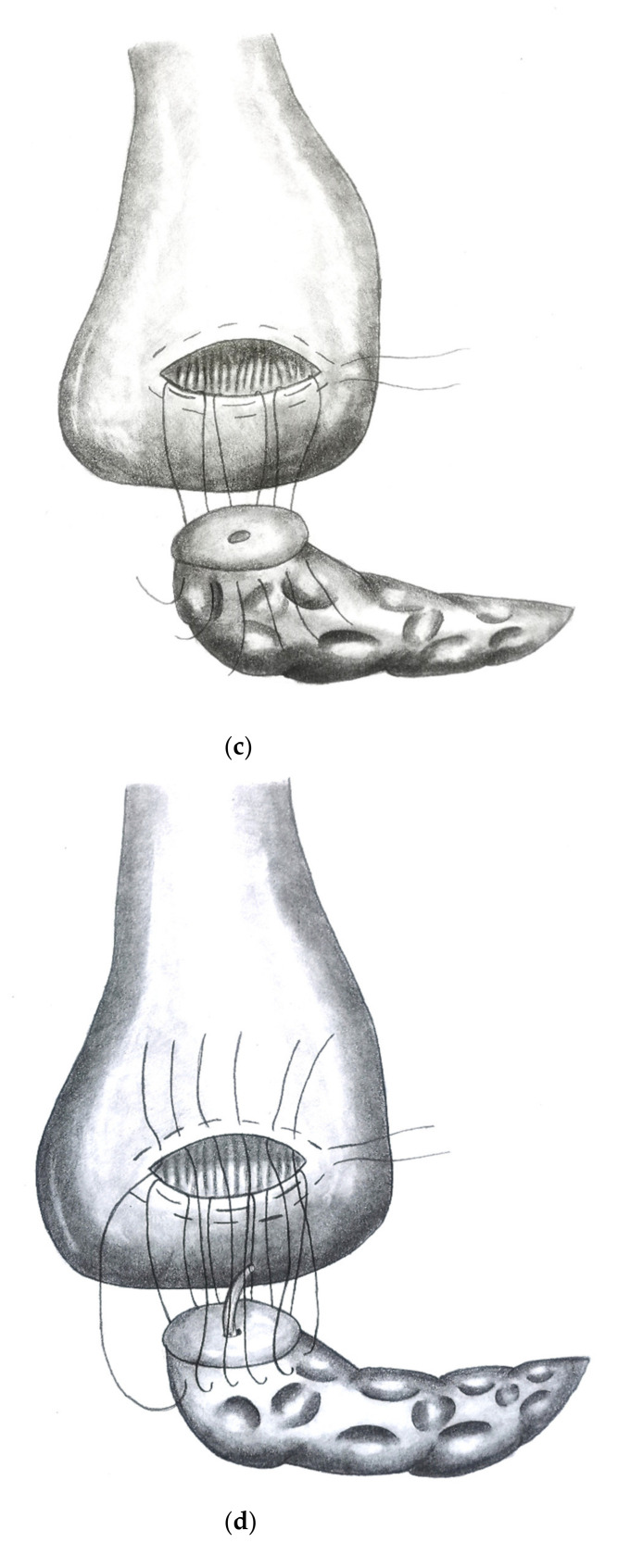

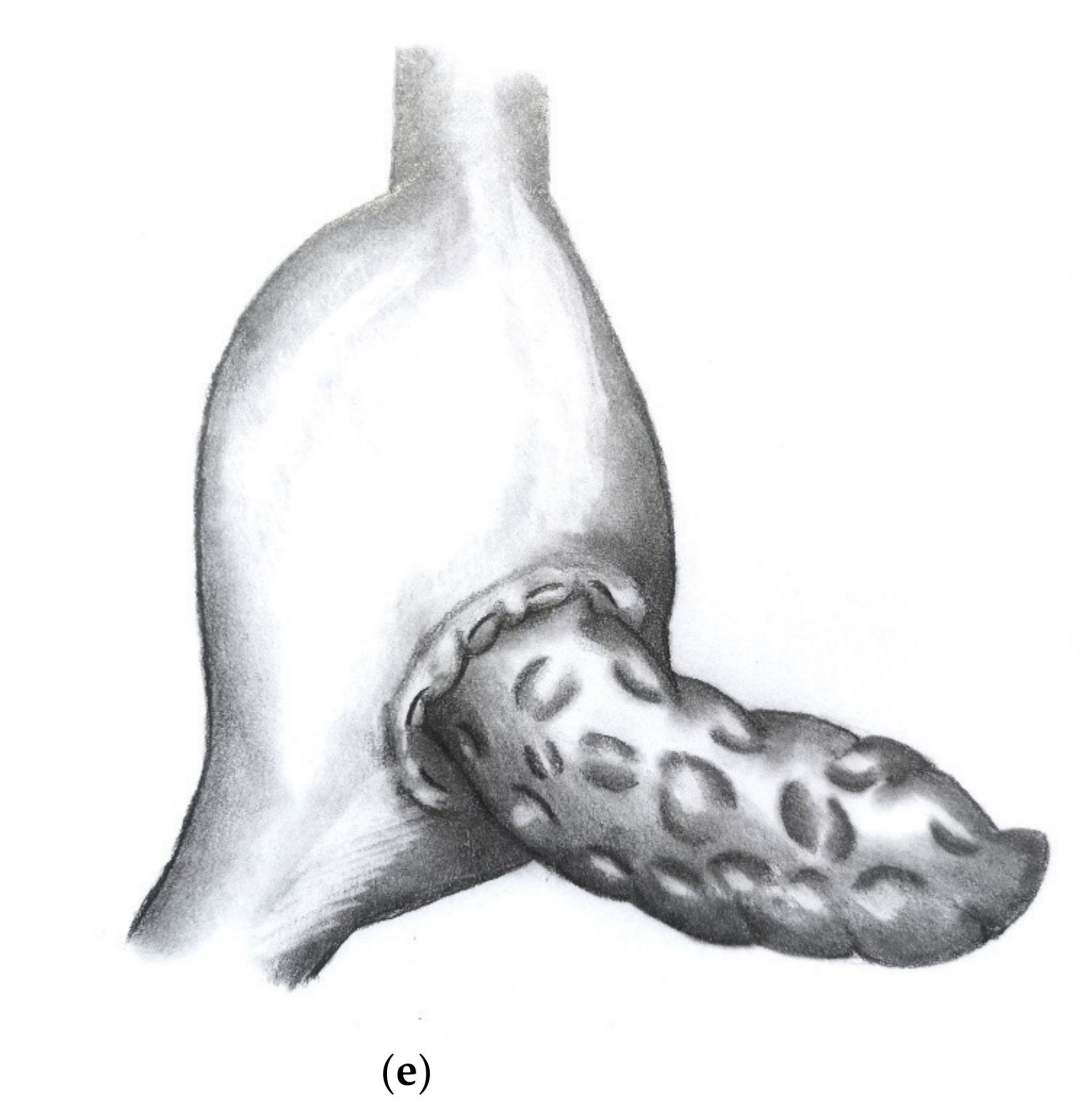
(**a**) Stomach lifted up exposing view on dorsal gastric wall with pylorus pointing upwards. An oblique incision is made to the dorsal gastric wall and a purse string suture (Prolene^®^ 4/0) is placed. (**b**) Three to four mattress sutures are then placed using double armed PDS^®^ 4/0 (MH1 needle) beginning on the cutting margin of the dorsal gastric incision. (**c**) Each string is then stitched through the parenchyma of the pancreatic remnant. (**d**) Each string is then stitched inside out through the opposite cutting margin of the dorsal gastric wall. (**e**) Finally, the pancreatic remnant is luxated into the posterior gastric wall and the mattress sutures followed by the purse string suture are tied.

Figure 2a–e show the implementation of a dioPG.

### 2.6. Postoperative Course

The postoperative course was standardised for all patients admitted to our unit for pancreatic surgery. Patients were admitted to a specialised intensive care unit and monitored for at least one day. Standardised postoperative evaluation included the close-knit measuring of blood glucose levels, inflectional parameters, blood cell count and level of lipase in the intraabdominal drainages as well as daily physical examinations. After exclusion of a POPF, drainages were removed. The nasogastric tube remained until an X-ray with contrast agents ruled out PG insufficiency and gastric emptying disorder due to swollen gastroenterostomy on day five after surgery and a gradual reintroduction from liquid to solid food was performed. A professional nutritional consultant and a diabetologist advised each patient by means of developing exocrine and endocrine pancreatic insufficiencies. If applicable, a specialised tumour board discussed each case after a histopathology determined malignancy.

### 2.7. Statistics

Data were processed using SPSS version 25.0 (IBM, Armonk, NY, USA). Two-tailed Pearson’s chi-square test and Fisher’s exact test were performed on categorical and ordinal scaled data. Two-samples-t-test was performed on interval scaled parameters. Significance tests were two-sided, and *p* < 0.05 was considered to be statistically significant.

## 3. Results

### 3.1. Patients’ Characteristics

An total of 141 patients was included in this analysis. Of them, 49 underwent dioPG and 92 of them underwent vgPG in the time between January 2017 and December 2019. Basic parameters, such as sex, age, ASA-score, preoperative BMI and indication, did not significantly differ in both groups. A total of 48% of all patients (n = 68) were male with a mean age of 64 years (23–88 years). Out of all patients, 78.3% underwent resection for malignant tumour (n = 73.8), including pancreatic adenocarcinoma, distal bile duct carcinoma, duodenal carcinoma and sarcoma. Table 1 indicates the patients’ characteristics.

### 3.2. Perioperative Parameters

The postoperative resection state was comparable in both groups (*p* = 0.511) with an R0-resection rate of 63.5% (*n* = 66) in all patients. Operation time differed significantly in both groups (*p* = 0.04) with a mean of 297.5 min (162–437 min) in dioPG and 320.5 min (191–485 min) in vgPG.

### 3.3. Complications

The rate of overall complications did not significantly differ in both groups (*p* = 0.301). The morbidity rate for patients after vgPG was 71.7% compared to 63.3% in patients after dioPG. A total of 42.9% of all complications in the dioPG group and 54.3% of all complications in the vgPG group were defined as Clavien/Dindo ≥ 3a [12]. Complications, such as POPF, PPH, SSI, PG-insufficiency and insufficiency of the hepaticojeunostomy, were comparable in both groups as well as the reoperation rate, rate of interventions, 90-day readmission rate and in-hospital stay. Two patients (4.1%) died after dioPG within the first 30 days after surgery due to myocardial infarction and hypoglycaemia. Five patients (5.4%) died after vgPG in the first 30 days after surgery; only one died due to severe bleeding, while four died due to pulmonary complications, such as aspiration pneumonia and pulmonary embolism. In the dioPG group, three patients underwent reoperation: two due to insufficiency of the hepaticojejunostomy and one due to fascia dehiscence. In the vgPG group, 13 patients underwent reoperation: one due to severe intraabdominal bleeding, two due to insufficiency of hepaticojejunostomy, one due to a non-retrievable drainage, one due to postoperative ileus, one due to abdominal compartment, one due to PG-insufficiency, four due to wound healing disorders and two due to pneumothorax. However, significantly fewer patients developed DGE when undergoing dioPG compared to those who underwent vgPG (*p* = 0.036). Table 2 indicates perioperative parameters and postoperative complications.

## 4. Discussion

Because insufficient restoration of the pancreatic remnant correlates with morbidity and mortality, e.g., the development of POPF and PPH, the implementation of a pancreatico-enteric anastomosis remains, even 130 years after the performance of the first partial pancreaticoduodenectomy, still the ‘Achilles’ heel’ in pancreatic surgery. However, although the incidence of POPF decreased, the related mortality remained at 1% in the last decades, whereas the mortality related to grade C fistulas is still around 40–50% [13]. The occurrence is not only related to an insufficiency of the pancreatico-enteric anastomosis but likewise to injured tissue around the cutting surface, needle channels or injuries to the tissue due to cutting sutures [14]. Several risk scores for the appearance of POPF have been proposed. The most commonly used fistula risk score (FRS) includes BMI, gland texture, duct diameter, intraoperative blood loss and pathology [15]. An alternative risk score (aFRS) was proposed in 2019, including only BMI, duct diameter and tissue texture [16]. Up to a quarter of all restorations are done by PG [17]. Several studies did not detect a difference in the occurrence of POPF in PG or PJ [18,19,20]. There are several factors to be considered for reconstruction after PD. Independent risk factors for the development of POPF are parenchyma texture (softer tissue is related to a higher POPF incidence), avoidance of tension, number and tightness of sutures and sufficient blood supply [21]. Thus, invagination type PG is more often used than a duct-to-mucosa type PG. Invagination covers not only the pancreatic duct but also the cutting surface and transpancreatic placed suture channels. With this analysis, we provided the dioPG (Charité-PG) as a new technique for invagination type PG reconstruction after open PD and compared it to the vgPG as performed in our centre. There was no significant difference in both groups for the occurrence of POPF, PPH, overall morbidity and mortality. Thus, it appears to be a feasible and safe reconstruction technique which led to a significant shortage of operation time in our centre.

Significantly fewer DGE occurred after dioPG. As DGE is not life-threatening in the first place, it causes a prolonged in-hospital stay, decreases life quality and keeps patients from sufficient recovery, which may lead to issues in adjuvant treatment. It more often appears in patients with PG than in PJ, though it is seldom a primary phenomenon; however, it has a strong association to intraabdominal complications such as the appearance of abscesses and the development of POPF and PPH [22]. Omitting of an additional ventral gastrotomy may cause less irritation to gastric peristaltic. This study is limited by common biases, mainly due to its retrospective character. In our opinion, most relevant points for a successful PG are the tension-free reconstruction, full invagination to cover the cutting surface and transpancreatic suture channels, sufficient blood supply and avoidance of pancreatic juice impoundment with the use of a small rubber tube. Nevertheless, it requires experience.

## 5. Conclusions

We provide the Charité-PG as a new PG technique and a feasible option for reconstruction following PD that needs further assessment in order to also evaluate its applicability to minimally invasive procedures.

## Figures and Tables

**Table 1:** Patients’ characteristics.

Characteristics *N* (%)	All Patients (*N* = 141)	dioPG (*N* = 49)	vgPG (*N* = 92)	*p*-Value
**Sex *N* (%)**				0.896
Male	68 (48.2)	24 (49)	44 (47.8)	
Female	73 (51.8)	25 (51)	48 (52.2)	
**Age (years)**				
Mean	64	66.43	62.71	0.092
Minimum	23	46	23	
Maximum	88	88	85	
**ASA Score *N* (%)**				0.608
1	2 (1.4)	0 (0)	2 (2.2)	
2	74 (52.2)	24 (49)	50 (54.3)	
3	57 (40.4)	21 (42.9)	36 (39.1)	
4	1 (0.7)	0 (0)	1 (1.1)	
**BMI *N* (%)**				0.666
Mean	24.8	25.2	24.6	
Minimum	15.2	17.4	15.2	
Maximum	41.8	41.8	39.5	
**Indication**				
Malignoma	104 (73.8)	36 (73.5)	68 (73.9)	
Benign Lesion	37 (26.2)	13 (26.5)	24 (26.1)	

BMI = body mass index, ASA = American Association of Anesthesiologists, dioPG = dorsal incision only pancreatogastrostomy, vgPG = pancreatogastrostomy via ventral gastrotomy.

**Table 2:** Perioperative parameters and complications.

Characteristics *N* (%)	All Patients (*N* = 141)	dioPG (*N* = 49)	vgPG (*N* = 92)	*p*-Value
**R0 Resection State *N* (%)**	66 (63.5)	25 (67.6)	41 (61.2)	0.511
**Operation Time (min)**				
Mean	312.5	297.5	320.5	0.04
Minimum	162	162	191	
Maximum	485	437	485	
**Overall Complications *N* (%)**	97 (68.8)	31 (63.3)	66 (71.7)	0.301
**Clavien/Dindo Classification** ***N*** **(%)**				0.6
0	44 (31.2)	18 (36.7)	26 (28.3)	
1	14 (9.9)	4 (8.2)	10 (10.9)	
2	12 (8.5)	6 (12.2)	6 (6.5)	
3a	34 (24.1)	5 (10.2)	29 (31.5)	
3b	11 (7.8)	4 (8.2)	7 (7.6)	
4a	15 (10.6)	9 (18.4)	6 (6.5)	
4b	2 (1.4)	0 (0)	2 (2.2)	
5	9 (6.4)	3 (6.1)	6 (6.5)	
**POPF *N*(%)**				0.737
Biochemical Leak	5 (3.5)	2 (4.1)	3 (3.3)	
B	18 (12.8)	7 (14.3)	11 (12)	
C	2 (1.4)	0 (0)	2 (2.2)	
**PPH *N* (%)**				0.899
A	5 (3.5)	2 (4.1)	3 (3.3)	
B	12 (8.5)	5 (10.2)	7 (7.6)	
C	2 (1.4)	1 (2)	1 (1.1)	
**SSI *N* (%)**	22 (15.6)	7 (14.3)	15 (16.3)	0.753
**DGE *N* (%)**	28 (19.9)	5 (10.2)	23 (25)	0.036
**PG-Insufficiency *N* (%)**	7 (5)	3 (6.1)	4 (4.3)	0.644
**Insufficiency hepaticojejunostomy *N* (%)**	11 (7.8)	4 (8.2)	7 (7.6)	0.907
**Reoperation Rate *N* (%)**	16 (11.3)	3 (6.1)	13 (14.1)	0.153
**Intervention *N* (%)**	53 (37.6)	15 (30.6)	38 (41.3)	0.212
**30-day Mortality *N* (%)**	7 (5)	2 (4.1)	5 (5.4)	0.725
**90-day Readmission Rate *N* (%)**	12 (8.5)	4 (8.2)	8 (8.7)	0.914
**In-hospital stay (days)**				
Mean	19.79	18.37	20.54	0.261
Minimum	7	9	7	
Maximum	59	59	56	

dioPG = dorsal incision only pancreatogastrostomy, vgPG, pancreatogastrostomy via ventral gastrotomy, POPF = postoperative pancreatic fistula, PPH = postoperative pancreatic haemorrhage, SSI = surgical site infection, DGE = delayed gastric emptying.

## Data Availability

Data are available from the corresponding author on reasonable request.
